# Towards a social psychology of precarity

**DOI:** 10.1111/bjso.12618

**Published:** 2023-01-13

**Authors:** Clare Coultas, Geetha Reddy, Johanna Lukate

**Affiliations:** ^1^ School of Education, Communication and Society King's College London London UK; ^2^ Department of Psychology and Counselling Open University Milton Keynes UK; ^3^ Max‐Planck‐Institute for the Study of Religious and Ethnic Diversity Göttingen Germany

**Keywords:** coloniality, interdisciplinary, praxis, precarity, social psychology, transdisciplinary

## Abstract

This article introduces the special issue ‘Towards a Social Psychology of Precarity’ that develops an orienting lens for social psychologists' engagement with the concept. As guest editors of the special issue, we provide a thematic overview of how ‘precarity’ is being conceptualized throughout the social sciences, before distilling the nine contributions to the special issue. In so doing, we trace the ways in which social psychologists are (dis)engaging with the concept of precarity, yet too, explore how precarity constitutes, and is embedded within, the discipline itself. Resisting disciplinary decadence, we collectively explore what a social psychology of precarity could be, and view working with/in precarity as fundamental to addressing broader calls for the social responsiveness of the discipline. The contributing papers, which are methodologically pluralistic and provide rich conceptualisations of precarity, challenge reductionist individualist understandings of suffering and coping and extend social science theorizations on precarity. They also highlight the ways in which social psychology remains complicit in perpetuating different forms of precarity, for both communities and academics. We propose future directions for the social psychological study of precarity through four reflexive questions that we encourage scholars to engage with so that we may both work with/in, and intervene against, ‘the precarious’.

## BACKGROUND

**FIGURE A1:**
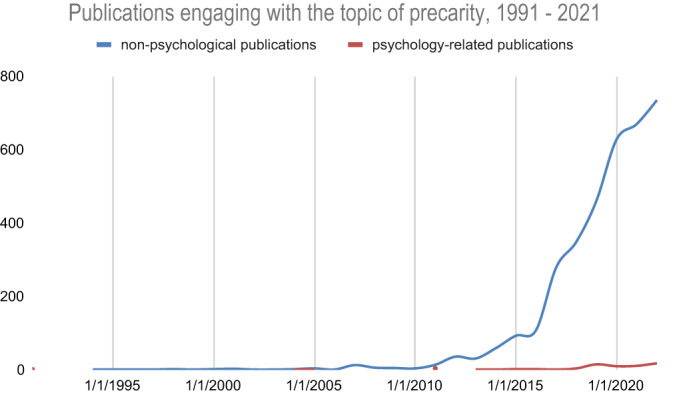
Graph displaying 3584 publications on the topic of precarity between 1991 and 2021. The blue line shows publications outside the field of psychology, with the majority of publications coming from sociology, geography and anthropology. The red line captures psychology‐related publications, whereby a relation to psychology was either established in the article title or the journal title. Based on this analysis, psychology‐related articles account for 1.8% of the publications on the topic of precarity (JML). *Data source:* Web of Science

The concept of precarity has garnered significant attention in the social sciences in recent years evidenced by a steep rise in publications on precarity since 2017, however, less so in the discipline of (social) psychology (see Figure A1). In this special issue, we look to explore what a social psychology of precarity could be and support the situating of social psychology in and amongst work being done in other disciplines, such as in geography (Philo et al., 2019), sociology (Schierup & Jørgensen, 2016) and anthropology (Wool & Livingston, 2017). Butler's (2009) definition of precarity is a common starting point in these literatures – as ‘that politically induced condition in which certain populations suffer from failing social and economic networks of support and become differentially exposed to injury, violence, and death’ (p. ii). At quite a surface level, ‘the psychological’ in this definition could be seen in terms of the suffering that people experience in precarity‐as‐a‐condition. However, psychology as a discipline is also implied, and incidental to the broader politics of Butler's conceptualisation of precarity; seen in the call to shift blame away from individuals who are experiencing precarity, towards more structural and politicized understandings of this condition, along with mobilizing collective action against it. Whilst this is a useful and important manoeuvre for scholarly work on precarity, the psychology that is implied herein remains rather reductionist and therefore ripe for discussion.

In this special issue, we work to challenge this reductionist view and highlight the legacies of social psychological work which have generated insights beyond the individualist understandings of suffering and coping that underpin ‘blame’ discourses. These include critical and community psychologies (Fine, 2012; Henriques et al., 1998; Kagan et al., 2011; Kessi et al., 2021; Teo, 2015), political psychology (Hammack & Pilecki, 2012; Howarth & Andreouli, 2017), liberation psychologies (Martín‐Baró, 1994), Indigenous (e.g. Māori psychology – Rua et al., 2022), and Africa(‐n), centred anti‐racist, decolonial and feminist psychologies (Kessi & Boonzaier, 2018; Malherbe et al., 2021). For example, studies examining how communication about unemployed people are framed (Okoroji et al., 2021) and on social representations of poverty (Chauhan, 2016; Chauhan & Foster, 2014) are reflections of critical social psychological research that treat suffering as relational and take into account the socio‐political and historical contexts that produce poverty. Rather than understand emotional distress as detached from the social context in which a person lives, Burgess and Campbell (2014), for instance, show how poverty, family, violence and HIV work together to shape how HIV/AIDS‐affected women in South Africa make sense of their emotional distress. And instead of victimographies, Hodgetts et al. (2014) advocate for psychologists researching homelessness to work in partnership with communities. Collectively, this body of work challenges the dominance of individualist understandings through (re)framings of the individual as embedded within political, economic, structural and historical contexts and relations of power and dominance, and can offer much to the politically‐ and structurally oriented understanding of precarity that Butler (2009) calls for.

We also look to open‐up explorations of what ‘the psychological’ can mean in precarity and highlight the potential of social psychology for enriching conceptualizations of precarity. One of our intentions is therefore to build upon work in other disciplines by exploring both how different conceptualizations of precarity weave throughout social psychological understandings of social phenomena, as well as how social psychology can be woven into existing knowledge on precarity, resulting in the co‐production of a rich tapestry of academic thinking on precarity in different social worlds. In her keynote at the 2015 conference for the International Society of Critical Health Psychology, Michelle Fine described precarity as ‘a profoundly psychological idea… [being] the sense of the predictability of the unpredictable, the experience of contingency and fear, [and how] the deep embodied sense of insecurity is… existential and affective, and it's in all of our lives’. Yet why has precarity as a concept been so underexplored and under‐theorized in the discipline? We posed this question in the Social Psychology Special Section of the British Psychological Society's annual conference in 2018 which looked to unpack the discipline's ‘crisis of relevance’ (Giner‐Sorolla, 2019). In a symposium titled ‘The Politics of Voice in Researching Individuals‐in‐Contexts of Precarity’, the three of us, with Nihan Albayrak‐Aydemir and Celestin Okoroji (all early career scholars), invited social psychologists to reflect on how the discipline and the methodologies it adopts, can both facilitate and hinder understandings of Selves in precarity. Benefitting from the single presentation format, we engaged in lively discussions with colleagues who attended the conference and many came forward to express their interest in contributing to an edited collection that would explore the role of precarity in social psychology. The time it has taken for us to develop this dialogue into a special issue is indicative of the precarity of our own positionalities as early career researchers transitioning into our post‐doctoral careers. These experiences speak to how precarity is embedded in the psychological as well as in psychologists' lives.

## SPECIAL ISSUE INTERVENTIONS AGAINST AND DIALOGUING (WITH) PRECARITY

In some ways, this special issue is an intervention *against* precarity. We recognize our editing of this special issue as early career scholars is an important action towards addressing the academic precarity outlined by Albayrak‐Aydemir and Gleibs (2022). We have, for instance, experienced academic precarity through the vehicles of fixed employment contracts and visa violences. Furthermore, whilst we have personally deeply benefited from the guidance of senior scholars, we situate ourselves in an academic system that has been seen to silence and punish early career scholars for articulating their views especially when critical of senior academics (see Peterson, 2022; Wellesley Alumni, 2022). We also recognize that special issue editorial teams often comprise of (or are wholly made up of) senior scholars who have dedicated their academic careers to the topic at hand, though exceptions to this unspoken rule encouragingly exist (cf. Amer & Obradovic, 2022; Salvati & Koc, 2022). This normative practice raises broader questions of academic freedom that reveal how systems of power and interlocking oppressions affect gender minorities, racialized scholars and international colleagues more intensely (Blell et al., 2022a). We are very appreciative of the *British Journal of Social Psychology* (BJSP) Editors Laura Smith and Stephen Gibson for this opportunity that allows us to contribute to pertinent debates in the field of social psychology, and the academy more broadly. It has also, undeniably, facilitated our own journeys out of more precarious engagements with the academy to more stable employment positions in environments that support our scholarship.

The precariousness of academic scholarship has been a running theme throughout the development of this special issue. Members of our editorial team, contributing paper authors and reviewers have moved countries, gained and left jobs and fixed‐term contracts, moved homes, fallen ill, lost and/or taken on caring responsibilities of relatives and welcomed and birthed new family members, all whilst living through a pandemic that has not affected everyone uniformly. Within academia, women and gender minorities of colour have been shown to experience the sharper end of the pandemic (Blell et al., 2022b), and incorporating an understanding of this reality into our praxis has been a conscious intervention on our part as special issue editors. This special issue has therefore been an exercise of slow(er) scholarship (Lau, 2019; Mountz et al., 2015) that could not have materialized without the understanding and commitment of all involved in this process. We would like to thank and recognize the work and support of the BJSP Editors, as well as Sharumathi Selvarajan, Vinubala Viswanathan, Akila Lakshmanan, Dinesh Mohan, Shreya Srivastava, Jernisha Jenifer Dhas, Hannah Wakley and other members of the Wiley Editorial Team in making this centring of relationalities possible in a publishing system that is not built with this in mind! We see this decision to engage in slower scholarship as a conscious action against academic precarity and invite future editors to grapple with academic pace and our roles in perpetuating academic precarity in academic scholarship.

As special issue editors, we view this slower scholarship as a means of *dialoguing* (with) precarity; how creating space for dialogues, relationship‐building and room for thinking has been key to supporting our explorations with the concept of precarity whilst also contending with aspects of precarity and precariousness in our own and others' lives and careers as mentioned above. Taking direction from the Readsura Decolonial Editorial Collective (2022a, 2022b), we have worked to operate as Co‐Editors of this Special Issue in more dialogical ways. Our editorial process involved first, reviewing all manuscripts internally within the special issue editorial team, then sending them off for external review to two or three reviewers. At least one of these reviewers was a social psychology scholar who had expertise in the subject area of the paper. The other reviewers were experts in the subject area but may have located their scholarship in other disciplines such as sociology, political sciences or human geography. After receiving all external reviews, the editorial team came together to review those reviews and put together an Editors' report. These steps were repeated at each stage of revision. We also held online discussions with authors and reviewers, looking to create and hold spaces for interactions between authors, reviewers and with ourselves beyond the standard review process. In doing this, we were not only working to subvert the automatization and distorted communicative practices that are common to academic publishing processes but also unsettle entrenched academic hierarchies which promote individualistic and competitive conceptions of knowledge production and academic critique. Instead, we looked to support the co‐conceptualization of social psychological knowledge on precarity, bringing together all scholars involved – editors, special issue editors, reviewers and manuscript authors. Nevertheless, these disruptive actions have, perhaps expectedly, resulted in a push and pull between all scholars involved. For whilst there were enormously generative aspects to this way of working, we do recognize that the time taken to convey our editorial engagements and the speed by which articles were then processed may have been new. Furthermore, we recognize that there is a safety in the faceless and formulaic approach to communication in academic publishing, and accordingly, our more dialogical and collaborative approach may have been discomforting for some scholars.

Another (discomforting) aspect of dialoguing (with) precarity relates to recognizing how disciplines themselves are precarity‐making. In formulating the boundaries between disciplines, and the foci of each discipline, scholars hold presumptions of security, control and consistency, which is counterintuitive to how everyday life is experienced. Rigidity between disciplines and within each discipline leads to *disciplinary decadence*, ‘the phenomenon of turning away from living thought…which takes the form of one discipline assessing all other disciplines from its supposedly complete standpoint’ (Gordon, 2014, p. 86), premised on [mis]conceptions of mastery and a ‘celebration of closure’ (Gordon, 2018, p. 239). Through this framing, disciplinary decadence could be said to breed a precarisation of disciplines, in that their siloization from one another not only fuels disconnections from everyday life but also the overlooking of solutions to societal challenges that do not fit neatly into disciplinary boundaries. Social psychology itself is no stranger to these dynamics; however, its precariousness as a discipline – namely its lack of unification – is also a point of discomfort for many in the field: its foundational purpose remains a topic of debate, centred around questions of whether [social] psychology is a natural science, along with widely varied stances on how to conceptualize and empirically explore relationships between the individual and society and between theory and practice (Boutilier et al., 1980; Dafermos, 2015; Parker, 2013; Stenner, 2015).

We offer a provocation: what if these varying discomforts related to dialoguing (with) precarity were understood as intrinsic to change and transformation? Viewing discomfort through the lens of generativity, not only opens up opportunities for intervening against precarity but also for addressing questions of social psychology's relevance to society. We take inspiration from Gordon's (2018) discussion on ‘living disciplines’ which, in contrast to disciplinary decadence, ‘reach to reality without attempting to capture, colonise or constrain it… [being] animated by a form of humility’ (p. 233). We invite readers to join us in *leaning into* feelings of discomfort, and to stay with the tensions, as we explore the generative potentials of dialoguing (with) precarity.

In working to constellate a social psychology of precarity, we are therefore not looking to articulate disciplinary distinctions and contributions, but rather strive to develop an *orienting lens* for social psychologists committed to working *with* other disciplines, concepts and knowledges[s], in attempting to address the societal challenges of the present day. This entails not only tracing the ways in which social psychologists are [dis]engaging with the concept of precarity but also exploring how precarity constitutes and is embedded within, the discipline itself. We view this as a ‘lively’ endeavour, that is, by nature, ongoing and incomplete. We also propose that this liveliness is fundamental to addressing broader calls for the social responsiveness of the discipline as a whole. We have encouraged contributing authors to consider this liveliness in their workings with precarity and have invited reviewers from other disciplines to support the fostering of this working *with* and *against* disciplinary boundaries. We would like to thank our community of reviewers (in alphabetical order) who have contributed insights from across the social sciences: Amena Amer, Anne Phoenix, Brett Neilson, Danilo Silva Guimarães, Farhan Samanani, Felicity Callard, Flavio Azevedo, Fouad Bou Zeineddine, Irini Kadianaki, Iván Cano‐Gomez, Jennifer Sheehy‐Skeffington, María‐Cecilia Dedios Sanguineti, Mark Griffiths, Matthew John Easterbrook, Michael Biddlestone, Michelle Ryan, Mindy Truong, Monique Guishard, Nancy Albhaisi, Nick Hopkins, Olivia Evans, Rochelle Burgess, Saloni Atal, Sam Johnson‐Schlee, Simon Goodman, Sita Venkateswar, Siwar Hasan‐Aslih, Sophie Zadeh, Valerie van Mulukom, Zeynep Demir, Zoé Samudzi and two anonymous reviewers.

This special issue thus stands as a collective venture towards consciously creating an unbounded understanding of precarity that offers a basis for continued conversations both within psychology as a discipline but also with other disciplines and communities outside of academia. It reflects the culmination of labour, thought, dialogue and action from scholars with different methodological, epistemological and socio‐political loci of enunciations and makes space for humility and exploration through acts of co‐theorizing. Doing so, we adopt a trans‐disciplinary approach that is transgressive as ‘it aims to put the psyche back into the social and material whilst keeping the social and material at play in the psyche’ (Stenner, 2015; p. 321). We will now trace some of the ways in which theorizations on precarity thread throughout the social sciences before introducing the contributions to this special issue.

## THREADS OF PRECARITY THROUGHOUT THE SOCIAL SCIENCES

Scholarship on precarity can be found throughout the social sciences, threading together theorizing and empirical research in sociology, anthropology, human geography, social psychology, political theory, queer theory and education philosophy. In keeping with our intention to listen to Gordon's (2018) call for ‘living disciplines’, we will not focus on the demarcation of disciplinary distinctions, but will rather explore thematically the various threads of precarity which connect and run between different disciplines. In doing this, we only incorporate social science scholarship which explicitly works with the concept of ‘precarity’. Nevertheless, it is important to recognize that aspects of precarity, along with the connected projects of unveiling structural inequalities and inequitable pasts, presents and futures are operating across a range of conceptual registers inclusive of decoloniality, intersectionality, abolition and Black and African feminisms, to name a few. Exploring the interconnections between precarity and these different conceptual fields is beyond the scope of this special issue. Instead, we seek to trace inter‐ and trans‐disciplinary dimensions to conceptualizations of precarity and have been interested to note that psychology as a discipline does often stand apart from this work.

Within psychological literatures, precarity and associated concepts such as insecurity, adversity and uncertainty, are in many cases only referred to as an aspect of context that is not necessarily theorized, for instance solely being named as a factor or backdrop to feelings of isolation, stigma, distress and suffering. Furthermore, the discipline is commonly called out by other disciplines in regard to its complicity in pathologizing people living in precarity. For example, individual‐focussed resilience and risk rhetoric is used to blame and explain why some people cope better than others, with Fine (2015) describing psychology as ‘the handmaiden of deficit damage discourse’. Nevertheless, as we have already stated, there are strands of social psychological research which contribute to workings *against* these individualistic and blanket representations and understandings of precarity, and we spotlight these strands in the thematic threads of precarity which follow, even if the concept of precarity is not explicitly used. We do this with the intention of bringing social psychology *into* inter‐ and trans‐disciplinary explorations of precarity. We are not suggesting that precarity should be used by these social psychological scholars, nor that precarity as a concept encompasses the breadth and depth of this work. Rather we see our roles as weavers, pulling together the different threads in theorizing on precarity. The themes outlined below are not exhaustive nor discrete, but rather serve the purpose of highlighting noticeable interconnections within and between different fields of study.

### Precarity as structuring relations

Perhaps the most commonly identifiable conceptualization and exploration of precarity relates to the ways in which it structures relations between people and place, albeit through different disciplinary lenses. Standings' (2011) sociological conceptualization of ‘the precariat’ draws attention to a new category of people whose lives are typified by uncertainty, but who too contribute to increasing instabilities within society, for instance, seen in the rise in political extremism. Much of the work in this ‘thread’ of conceptualizing precarity in the social sciences, explores the ways in which the dynamics of labour markets extend to everyday life, constituting unequal power relationships and varying opportunities for agency which are set by the formal and informal conditions of workplaces (Deshingkar, 2019; Strauss, 2018; Wee et al., 2019). Through this framing, precarity encompasses freelancers, sub‐contractors and flexi‐workers, but also illegal, seasonal and temporary forms of employment, all of which have impacts on housing opportunities, relationships of care and work within the home and access to credit along with the management of debt (Neilson & Rossiter, 2005). In more critical analyses, the importance of not collapsing these different forms of labour under one umbrella term is emphasized (Neilson & Rossiter, 2005; Schierup & Jørgensen, 2016). Neilson and Rossiter (2005) also stress the need to adopt a broader lens which acknowledges the precariousness of capital, and its control over global borders and labour mobility: ‘the current increase of precarious work in the wealthy countries is only a small slice of capitalist history. If the perspective is widened, both geographically and historically, precarity becomes the norm’ (no pagination).

With regard to (social) psychology, precarity in academia – seen in casualized and exploitative labour practices – is implicated in psychology's replication crisis (Callard, 2022) and inequalities in social psychological research and knowledge production more broadly (Bou Zeineddine et al., in press). Ryan and Haslam (2005) draw attention to how ‘glass cliff’ (i.e. high risk) leadership positions are often given to women, contributing to the precarisation of women leaders in the workplace. And Burgess et al. (2022), in their explorations of how racially minoritized young people in the United Kingdom experienced the COVID‐19 pandemic, identify employment precarity – for both youth and their parents – as a factor in young people's mental health. Groot et al. (2017) expand beyond the world of labour in their analysis of ‘the Māori precariat’ in New Zealand, also exploring precarities in relation to cultural expression and feelings of safety. This more expansive view of precarity (i.e. as a structuring context to relations) facilitates connections to a range of social psychological research. This includes analyses of decision‐making patterns in conditions of scarcity (Sheehy‐Skeffington, 2019), explorations of collective resilience, collective agency, psychosocial scaffolding and the social cure (Campbell & Cornish, 2021; Elcheroth & Drury, 2020; Jetten et al., 2017; Jovchelovitch & Priego‐Hernandez, 2013; Ntontis et al., 2021; Wakefield et al., 2019), as well as work looking at how insecurity and uncertainty are implicated in prejudice, victimization and intergroup dynamics (Greenaway et al., 2013; Këllezi et al., 2022; Leach & Livingstone, 2015; Nair & Vollhardt, 2020).

### Precarity as process

The inherent temporalities and processual makings of precarity are another thread which weaves throughout the social sciences. In a special issue on precarity in *Geoforum*, the editors expressed how across all eight papers there was an alertness ‘to how precarity is made, a processual becoming rather than a timeless structural property, and to how precariousness amounts to what might be cast as “a fragile assemblage of fragility”, contingently spun together from filaments of thought, feeling, practice and things (human and non‐human, material and virtual)’ (Philo et al., 2019, p. 150). The political theorist Lorey (2015) identifies three interconnecting dimensions to ‘the precarious’, each of which indicate different forms of relationalities and dynamics: *precariousness* which reflects a socio‐ontological ‘being‐with’ (cf. Nancy, 2000) other precarious lives (i.e. living existence); *precarity* that designates precariousness, through processes of othering, as a condition of inequality; and *governmental precarization* which draws attention to how the discriminatory logic of security (that is dependent on the continual othering of vulnerability/insecurity) is central to liberal (self‐)governing. Lorey (2015) also identifies how precarization is currently undergoing a process of normalization in neoliberalism, which does not reflect an equality in insecurity, but rather how ‘precarization becomes a normality with new inequalities. The imaginary centre of the normal is not simply threatened, nor is it merely unsettled. Instead it becomes itself increasingly insecure and threatening’ (p. 67–8), seen in rising preoccupations with domestic security and border controls. This logic of security(/vulnerability) connects with Fine's (2015) call to interrogate the psychology of elites, and what she describes as the ‘aggressive appetite for space, power, and control’, which is inherent to expansionist notions of societal progress, dependent on exploitative labour, border technologies and ecological degradation. Wool and Livingston (2017) also suggest that much social theorizing perpetuates the moralizing that is inherent to (neo)liberal narratives about progress; how visions and orientations towards a ‘vital’ future are constructed as a ‘false dilemma’ against ‘the unproductive dead ends of a toxic or melancholic present’ (Wool & Livingston, 2017, p. 5). Khan (2022), likewise, argues that utopic thinking must be ‘concrete’ (cf. Muñoz, 2009) and envisioned from positions of precarity.

Social psychological analyses of precarity‐as‐process can be seen in Kesisoglou et al.'s (2016) psycho‐discursive analysis of how young people in Greece construct agency, subjectivity and precarious work conditions. More broadly, a range of social psychological work using diverse conceptual lenses could be connected to this thread of precarity. This includes Joffé's (1999) theorizing on risk and ‘the Other’, along with explorations of politics, precariousness and transformations in (social) identities and collective action (Phoenix et al., 2017; Jovchelovitch et al., 2020; Albhaisi, 2022; Guimarães, 2021), and also meaning‐making in relation to dimensions of precarity such as poverty (Chauhan, 2016), unemployment (Okoroji, 2020; Okoroji et al., 2021; Pultz & Hviid, 2018) and suffering (Atal & Foster, 2021). Social psychology therefore has much to offer to analyses exploring how inter‐relations between Selves, Others and social knowledge are implicated in processual makings of precarity.

### Precarity as a hope‐ful/‐less place

Another identifiable thread relates to the affective dimensions – both hoping and hopelessness – to navigating the temporalities of precarity broadly outlined above (e.g. human existence, self‐other relationalities and societal shifts and changes). Srila Roy's (2019) ethnographic study of women empowerment workers in India – who do community and gender development work in localities – illustrates how interventions aimed at supporting ‘the precariat’ paradoxically produce precarity. Namely, how the ‘new capacities to aspire’ (p. 394) that are promoted by these organizations and ‘development’ programmes reflect ‘structurally unrealisable’ classed and gendered futures. Fine (2015) also discusses the ‘emotional precarity’ related to the insecurity of trusting and hoping again, after the collectivized disappointments of ‘the tomorrows’ promised never coming to be, and gives the Obama administration (e.g. see Darity Jr., 2016) and Arab Spring (e.g. see Gutman, 2022) as examples of this. Research in the fields of anthropology and human geography has been exploring the ways in which people function in contexts and conditions where life is disconnected from any hope for a better tomorrow, what Wool and Livingston (2017) describe as ‘collateral afterworlds’; places and communities where the precariousness of life is exploited, and politicized renderings of suffering and struggle made meaningless (Shaw & Byler, 2016). Philo et al. (2019) also discuss ‘psychic topographies’ in regard to the connections between place and living ‘on the edge’/*on edge*, encapsulating the stresses and anxieties and ‘unliveability’ of ‘deprived corners’ of the world. Johnson‐Schlee's (2019) ethnography of a soup kitchen in South London, for instance, illustrates how service users deploy conspiracy theories as a means of resisting and circumventing societal and political discourses which stigmatize and exclude them.

Wool and Livingston (2017) also draw on queer theorizing and interventions on the ‘antisocial’ to draw attention to the moralizing that is intrinsic to narratives about sociality and social integration. By exploring how people inhabit this liminal space ‘in‐between sociality and decay’, they work to recognize the different forms of attachment that can constitute life and hope. Joronen and Griffiths (2019a) identify hope(ful waiting) in ‘hyperprecarious sites’ in Palestine as a processual time–space made‐up of everyday practices that refuse and disrupt the ‘binds’ of Israeli occupation. Brown's (2019) social psychological work looking at ‘vital spaces’ and ‘life‐spaces’ (Reavey et al., 2019) in relation to mental health and recovery could also be connected to these considerations of constellating hope within hopelessness. Hasan‐Aslih et al. (2019) alternatively, question the utility of hope in collective action and illustrate how ‘hope for harmony with the outgroup’ can in fact undermine the ‘constructive tension’ needed to mobilize disadvantaged groups towards fighting for equality.

### Precarity as generatively ambivalent

The last thread that we would like to highlight explores in more depth how the ambivalence seen in the previous thread (precarity as a hope‐ful/‐less place) extends beyond the affective, holding generative potentials; how precarity can be both ‘politically debilitating’ *and* ‘ontologically productive’ (Joronen & Griffiths, 2019b). Human geographers, for instance, highlight how precarity is built into the materialities and socialities of present‐day cities – ‘precarious urbanisms’ – with the gentrifying of areas being built upon the rebranding of insecurity and ephemerality as positive, such as in labour economies (e.g. ‘pop‐up’ restaurants, shops etc.), as well as in housing (e.g. property guardianships; Harris, 2020; Philo et al., 2019). In the field of education philosophy, Zembylas (2019) discusses the ambivalence of precarity in regard to its ‘multidirectional potential’, in that it can both result in violence and breakdowns in communication across differences, but can also foster connections and collaborations. In his consideration of a ‘critical pedagogy of precarity’, Zembylas (2019) identifies the ambivalence and differential distribution of vulnerability in society as a potential enabler for supporting learners to historicise (rather than psychologize) societal oppressions and injustices and develop understandings of the politicization of precarity: ‘students and teachers may be moved towards ethical responsiveness and political action when the ambivalent character of precarity is taken into account’ (p. 97). Lorey (2015) too views precarity as a potential starting point for activism, and certainly the increasing precarisation of life, work and the planet have become core principles around which collective action is being organized. Based on her work with women of colour activists, Emejulu (2022) states that:

ambivalence can be a way to misfeel and an emotional process by which solidarity can be built and sustained. There can be deep satisfaction and pleasure in ambivalence, as this emotion need not always be understood as a mode of internal conflict. Rather, ambivalence can be understood as a moment of contemplation, of recuperation, a state of critical self‐reflection, a pause, a hesitation before meaningful action can take place…Ambivalence then is that precarious balance between understanding the forces arrayed against you that cause deep harm, and the possibilities, the longings for self‐tending and pleasure in community with like‐minded others

Nevertheless, an ambivalence remains in how we as academics support activists and struggles for social justice. Calls to decolonize the social sciences (Tuck & Yang, 2014), for instance, highlight the complicity of academic research in constructing and reifying ‘the vulnerable’ through the neglect of the dominant forces which structure this vulnerability. Attending to the ethico‐politics of collaborative transformative praxis is central to community, critical and Indigenous psychologies. There are, of course, no ‘easy fixes’, however, suggestions for attending to the ambivalence of power relations in (social) psychological research include: a reimagining of the American Psychological Association's (APA) Ethics Code (Guishard et al., 2018); adopting a dialogical epistemology (Zadeh, 2017); and engaging with a decolonizing praxis, reflected through a range approaches, inclusive of the centring of critical, socio‐historically situated, and reflexive engagements with the *effects* of research (Kessi & Boonzaier, 2018), practicing refusals (Coultas, 2022; Tuck & Yang, 2014) and a range of orientations for disrupting the coloniality of power (Fernandez et al., 2021).

We propose that this conceptual landscape of precarity in the social sciences presents exciting possibilities for a social psychology of precarity, illustrating both silences and opportunities for reflection and expansion in social psychological research. Fine (2015) also describes precarity as a ‘creative space’ which supports collective rethinking and reworkings of presumed stable categories, identities and structural alignments. We invite readers to join us in this creative exercise of not only considering how we can better work with and support communities living in conditions of precarity but also in our [re‐]opening‐up of space for questioning what ‘the psychological’ can mean, both within the discipline, but also as a means of building networks and connecting with other disciplines and sites of collective action.

## CONTRIBUTIONS TO THIS SPECIAL ISSUE

The special issue holds a total of ten papers. This introduction is followed by eight contributing papers and an invited epilogue by Michelle Fine (2022) which provides commentary on the special issue in addition to broader provocations for the field of (social) psychology, highlighting both the potentials and tensions of working with what she describes as ‘prec(ar)ious knowledge’. We will now provide a brief overview of the eight contributing papers before reflecting on the potentialities of ‘the social psychological’ in workings with/in precarity.

The ways in which academia and [social] psychology as a discipline are precarity‐*making* is the focus of four of the eight contributing papers in this special issue; however, they approach and conceptualize this issue in different ways and from different positionalities. Albayrak‐Aydemir and Gleibs (2022) adopt a position ‘inside’ social psychology, drawing on social identity theory, system justification theory and the inequality regime framework from sociology, to illustrate how precarious conditions in academia are [re]created through sets of practices, highlighting how we both ‘do’ precarity and can ‘be’ made precarious in academia as an organizational setting. They call for changes, not only in how we do research but also in how we identify as academics. Three of the papers – Hakim et al. (2022), Rua et al. (2022), and Reddy and Amer (2022)‐ adopt a critical position against hegemonic [social] psychology, broadly defined by Adams et al. (2018) as the practice of imposing a particular regime of cultural knowledge (e.g. modern individualism) as the global universal standard, and the coloniality which is often embedded within this. In Reddy and Amer (2022), the operationalisation of hegemonic psychology in the neoliberal university is mapped, seen not only in the coloniality of knowledge and presumptions of universality but also in how we document and disseminate knowledge, along with the ways in which social psychological theories and methods remain complicit in the upholding of whiteness as a system of power. They draw on a range of critical theorizing in their call for a reimagining of the university, most specifically related to the ways in which we can better foster relationalities, and make space for engagements with more diverse knowledge forms in research, teaching and also academic organization and experience.

In the paper by Hakim et al. (2022), the illustration of how hegemonic psychology is precarity‐making relates to the community of study (as opposed to academia itself). Through a critical review of experimental research on the Palestinian context in mainstream social psychology journals, this piece highlights how the complete non‐recognition of settler‐colonialism in this body of work naturalizes and contributes to Palestinian precarity. The study unsettles presumptions of researcher objectivity in this methodological approach, drawing attention to the political position that this non‐recognition of settler‐colonialism supports. As the authors articulate, there is no ‘view from nowhere’, and a lack of acknowledgement and engagement with this in social psychological research can have grave implications for the communities that we work with. The final paper which tackles this issue of social psychology being precarity‐making – Rua et al. (2022) – highlights the work that is being done ‘outside’ of mainstream social psychology, most specifically, in collective resistance against the "hegemonic tendency to conduct research *on* rather than *with* communities", as well as the dominance of the Western mind/world dualism and atomistic approach to studying the social world. The authors outline their body of work which draws together Māori cultural perspectives and assemblage theory towards highlighting both the dynamism in, and multi‐layered nature of, formations of the Māori precariat, as well as the potentials for doing research in more humane ways, centred around relational ethics and understandings of care. These four papers together raise important points for reflection in regard to how we do research and academia, drawing attention to the various ways in which our relationalities with others – both within and outside of the academy – can constitute complicities in ongoing oppressions and injustices.

Another two articles in this special issue outline in more methodological depth the importance, but also complexity, of analysing precarity using quantitative methods. Whilst each paper approaches the exploration of precarity differently, they highlight the potential of this area of work for expanding our analyses of societies beyond the commonly used measures and analyses of socioeconomics and class, in both cases holding social justice implications. Schmitt et al. (2022) look at how ‘flexible capitalism’ which expects people to be flexible in their use of time and space to cater to new labour practices, places extra stresses on lower socioeconomic groups in the United States, using the ‘time–space distanciation’ variable to analyse this. Not only does this study work to make visible the structural‐cultural contexts of neoliberal and flexible capitalism, it also challenges more mainstream individual‐focussed studies of decision‐making in conditions of scarcity which have pathologised the ‘present‐oriented mentalities’ of lower social class groups. Namely, the paper highlights the need to recognize how ‘mentalities’ and decision‐making are the products of structural affordances, and the particular stresses posed by both temporal and spatial aspects of precarity. In Adam‐Troian et al. (2022), precarity is recognized as ‘stemming from objective conditions of affiliative and economic deprivation’. Building on earlier work that shows a link between economic inequality and the endorsement of conspiracy beliefs, the authors offer a multi‐site study looking at the relationship between subjective experiences of precarity and the endorsement of conspiracy beliefs. In discussing the implications of this research, the authors also raise the point that increasingly much of modern life is typified by a sense of ontological insecurity and existential threat across all socioeconomic levels, and identify the need for work that enables analyses of precarity in more expansive ways.

The final two contributions to the special issue speak to this methodological call for exploring precarity as something that is experienced by all. In Mahendran et al. (2022), different models or manifestations of ‘psychological precarity’, identified as the sense of threat and insecurity that is inherent to people's identifications with ‘the global’, is explored through the development of an innovative methodology. Participants are given the ‘hyperagentic’ position of choosing how or if to construct borders on maps of the world, generating insight into the different relationalities (with both human others and more‐than‐human worlds) which constitute everyday understandings of precarity. Lastly, in Lukate (2022), the precariousness of address is considered through a phenomenological analysis of interview data in which diasporic women of African descent describe being called ‘white’ when they travelled to the African continent. Expanding on this, Lukate (2022) unpacks the analytic and ethical complexities of working against hegemonic practices of categorization in research and reflects on the ways in which researchers can be complicit in the re/production of social categories that ‘name, see, and know’ research participants through the colonial logic of Western epistemes. Both of these papers connect with Hakim et al. (2022) in highlighting how researchers can be complicit in coloniality and discriminations, purely through the ways in which we represent social phenomena, and the language and terminologies that we use.

## WEAVING SOCIAL PSYCHOLOGICAL THREADS FOR WORKING WITH/IN PRECARITY

As special issue editors, we propose that the contributions to this special issue present two core opportunities for weaving social psychological threads into workings with and within precarity. First, the papers highlight an outward focus pertaining to how we can use social psychological theorizing and methods to illuminate the role of the social psychological in understanding precarity. In discussing this, we highlight some of the interesting extensions that the contributions to the special issue offer to social science theorisations on precarity. Second, the papers highlight the importance of an inward focus that works to reveal how social psychology engages with and perpetuates precarity; how the ways in which psychology is practised, taught and used in research can unwittingly contribute to the precarisation of people living with/in precarity. Viewing the contributions collectively, we articulate a position that a social psychology of precarity needs to combine both of these foci – inward and outward – to effectively contribute to the study and alleviation of precarity.

### Looking out

The special issue highlights the diversity of social psychological tools for developing understandings of precarity. The contributing papers draw on a range of social psychological concepts, theories and methodological approaches, including social representations theory (Mahendran et al., 2022), social identity theory and system justifications theory (Albayrak‐Aydemir & Gleibs, 2022) and time–space distanciation (Schmitt et al., 2022) and utilize quantitative (Adam‐Troian et al., 2022; Schmitt et al., 2022), qualitative (Hakim et al., 2022; Lukate, 2022; Mahendran et al., 2022), and participatory methods (Rua et al., 2022; Fine, 2022) to study precarity. More broadly, the contributions to the special issue highlight some interesting extensions to social science theorizations on precarity, drawing attention to questions of ontology and differential experiences in relation to time and positionality.

#### The dynamics of self‐other/‐world relations in becomings of precarity

Whilst subjectivities and affective dimensions are linked to the processual nature of precarity in social science theorizing, papers in this special issue explore the dynamics of these processes in depth. Adam‐Troian et al. (2022) and Mahendran et al. (2022) draw attention to the ways in which (different formulations of) precarity, experienced as ‘threat’, shape societal relations and political decision‐making. Mahendran and colleagues (2022) further expand this view of societal relations through their use of the migration‐mobility continuum (which locates all humans on a scale of migration) in exploring the different ways in which people make sense of, and might seek to change, our bordered world. Together with the Kaupapa Māori approach to social psychology articulated by Rua and colleagues (2022), these authors call for a worldview in which selves are recognized in relations which extend beyond human Others, and how these Self‐Other‐World relations shape understandings and experiences of precarity. Lastly, Lukate (2022) explores the precariousness that is inherent to processes of identification with/by selves and others through her analysis of racialised categorizing in her study of women travelling to the African continent. She identifies this precariousness by expanding on the various [aspects of] ‘Selves’ and ‘Others’ who are in a ‘four‐way conversation’ in processes of racialization: the interviewee; general/imagined African Others; the interviewer; and the interviewer's culture‐specific inner eyes. As a whole, these papers provide detailed insights into the dynamics of living and being in/with precarity, and too the psychosocial processes which could be at play in constituting the ambivalent character of precarity – its connections with both hope and hopelessness, its potential to instigate conflict/protectionism as well as collaboration – with the recognition that there is a precariousness to all our existences.

#### Discontinuities and disjunctures in precarity as process

A number of the papers extend beyond discussions about the temporal nature of precarity by highlighting the ways in which precarity structures differential experiences in relation to time and vice versa. In Reddy and Amer (2022) and Albayrak‐Aydemir and Gleibs (2022), precarity is linked to shortages of time, and how this fosters extractive and transactional relationships in research and the academy more broadly. Schmitt and colleagues (2022) identify precarity as the ‘darker side’ of ‘flexible capitalism’ which unbinds and stretches time and space through globalization, credit‐based finances and technologization. In this framing, precarity is conceptualized as a ‘continuous potential condition of risk’ that is objectively greater and more stressful for lower socioeconomic groups. Namely, ‘societal flexibility’ is reliant on technologies and labour practices that structure inequities and disjunctures in relation to experiences of time[−space]. Lastly, Fine (2022) discusses her creative research with immigrant students of colour in a US public school, in which the discontinuities of time, and the unpredictability of futures *in* precarity are poignantly illustrated. One student depicts this by drawing a clock with lots of question marks, whilst another draws a yellow brick road that people fall off after failing tests or encountering flying monkeys, all the while not knowing what, or if anything, is at the end of the road (Fine, 2022). Across these analyses, we can see how distortions and disruptions of time are a driving force in precarity *as* process. This not only provides much needed nuance to social science theorizing on teleologies of societal progress but also connects with broader anti‐capitalist theorizing (e.g. on ‘crip time’ – Lau, 2019) which identifies time – not only its reclaiming but also the need for collective mobilization in reframing relationships between time and productivity – as fundamental to movements against inequalities and exploitation.

Whilst we have not sought to identify a declarative conceptualization of ‘the social psychological’ in precarity, the papers in this special issue do offer glimpses of how precarity can be expressed across different social psychological registers; from more mainstream approaches, to critical, political, Indigenous and decolonial psychologies. They illustrate the manifold ways in which precarity can be understood as a profoundly social psychological concept, and too, the varied ways in which the core axiom of social psychology – interactions between people *in* contexts – is approached. Together, these papers provide insight into the workings of compounded insecurities, and the vulnerabilities that arise when multiple forms of marginalization (such as race, class, gender and displacement) intersect. Furthermore, they offer insight and points for reflection on how social psychologists can engage with the complex politics of current social crises.

### Looking in

All of the articles in the special issue have also invited readers to look within the field to understand our limitations in understanding precarity and our complicity in perpetuating it. Grounding social psychological work *in* contexts means not only reflecting on the particularities of the immediate context surrounding our research but also acknowledging our own social, political and cultural positionalities as we strive to survive the spread of ‘precarity capitalism’ (Azmanova, 2020) that operates within ‘imperialist white supremacist capitalist patriarchy’ (hooks, 2004, p. 17). In this light, Reddy and Amer (2022) offer a ‘sobering capture of the bleak state of affairs in whitestream psychology’ using a six point lens of precarity. Fine (2022) raises ‘friendly questions’ on how the focus of social psychological research on people experiencing precarity supports the continued obfuscation of the systems and machinations by which precarity is perpetuated. The politics of the knowledge produced in social psychology, seen through the different perspectival gazes and the roles that social psychological researchers take when conducting research, are also illustrated in the special issue. Within this context, articles discuss the power differentials that exist when social psychologists engage in exploitative relationships with their participants (Reddy & Amer, 2022; Rua et al., 2022), when scholarship from those experiencing the very violence that is being researched is strikingly absent (Hakim et al., 2022) or when social psychologists uncritically engage with commonly used social categories (Lukate, 2022). These revelations are important especially when social psychologists are focused on addressing the WEIRD‐ness of the discipline, as the papers carefully highlight the politics and ethics of working with marginalized and Indigenous communities. The limitations of quantitative methods in fully capturing the complexities of precarity – but too, the importance of work aimed at addressing the ways in which the frequented use of indicators and variables obfuscate precarity – are also revealed (Adam‐Troian et al., 2022; Schmitt et al., 2022). Such revelations also unmask the epistemological violences of problematising and interpreting the Other as inferior (Teo, 2010), along with epistemic violences related to elevating particularly positioned knowledges as the general standard, and imposing models inappropriate for local realities (Decolonial Psychology Editorial Collective, 2021). These different forms of violence then feed into the precarization of individuals. In sum, these contributions invite us to acknowledge the role that social psychology, and in particular its common methodologies, theories and ways of engaging with the ‘researched’, have played in perpetuating precarity.

A number of the articles offer proposals for countering the complicitous legacies of social psychology. Despite inroads being made to trouble the individualistic focus in social psychological workings with/in precarity, it is not surprising that issues concerning precarity such as poverty remain predominantly theorized and studied as a given aspect of context and more worryingly that solutions to poverty are being offered at the individual level. By inviting us to think beyond the human level, Mahendran et al. (2022) and Rua et al. (2022) elevate the often micro‐level focus of social psychology to a theorizing that encourages us to reflect on how the individual is part of a larger assemblage of world‐making experiences. Addressing the field's limited understanding of ethics of care, Reddy and Amer (2022) offer four political‐personal intentions that social psychologists can undertake to uproot precarity within the discipline. Calling for the field to be more reflexive with regards to how research is being framed and participants are being named, Lukate (2022) invites us to engage with the precariousness of address that privileges hegemonic Western ways of framing and naming research and its participants. By revealing the coloniality of knowledge embedded in the social psychological research on Israeli‐Palestinian relations, Hakim et al. (2022) invite us to re‐think and resist actions that weld more closely material and psychological colonizations. Finally, Albayrak‐Aydemir and Gleibs (2022) call for broad systemic change in academia to redress wage gaps, unequal power relations, unmanageable workloads and hostile academic environments. These proposals invite us to engage in actions that counter the epistemic and epistemological violence that can be inflicted when researching individuals in contexts of precarity and engaging in social psychological scholarship more broadly.

## WHERE TO FROM HERE? FUTURE DIRECTIONS

The aim of this special issue was to mobilize thinking towards a social psychology of precarity. The contributing papers illustrate the pluralities in social psychological thinking, which we view as a strength, in keeping with Gordon's (2018) discussion on ‘living disciplines’. The contributions of reviewers from different disciplinary standpoints have been fundamental to maintaining a view to this liveliness. Nevertheless, whilst this collective endeavour at dialoguing both *with* and *about* precarity has been enormously generative and enriching, disparities related to multiple intersecting axes of marginalization have also been revealed, in terms of who has been able to participate, in what capacity, along with how the knowledge of ‘others’ has been engaged with. The different structurings and differential experiences of precarity in academia is an issue that is discussed by many of the contributing authors, and we felt it important to also be open about how the making of this special issue was not immune to these dynamics. Therefore in considering ‘where to from here?’ we do not seek to consolidate ‘the social psychological’ in the study of precarity, nor limit the relevance and potential of the precarity concept to a number of social psychological areas of interest. Instead we pose a series of reflexive questions that we hope will support social psychological scholars to engage with the tensions and un(der)explored threads of precarity that remain. These questions are not exhaustive, and we make no claims to their novelty – in fact, many of them stem from the more critical legacies of social psychology and conceptual registers that we have discussed herein. Nevertheless, we do suggest that these four reflexive questions are fundamental to any social psychological endeavour at dialoguing with and about precarity.

### Who is absent from our analyses?

This relates to both who are being studied as well as who are doing the studying. In our call for papers, we invited contributions working with/in understudied contexts and populations (e.g. marginalized communities in the Global South, refugees, elites, etc.), yet these are notably absent in this special issue. Furthermore, the contributing authors are predominantly located in the Global North, whilst they may themselves be from the Global South. The paucity of Global South scholarship, along with research looking at what might be considered the extreme spectrums of precarity (i.e. precarity in the Global South as opposed to analyses of elite ‘precarity‐makers’), is a significant limitation of our collective exploration of precarity. As articulated by Bou Zeineddine et al. (in press) together with contributions to this special issue (Albayrak‐Aydemir & Gleibs, 2022; Reddy & Amer, 2022), we need to be putting more active focus towards revealing the ways in which academic precarity, along with geopolitical dominance and exclusions in the formation of academic networks, structure how and why knowledge is (not) produced. If we are to meaningfully engage with precarity, it is imperative that we not only politicize our work but also the ways in which we work.

### How might we be complicit in colonizing, exclusionary and silo‐izing knowledge practices?

This speaks to how we negotiate both the politics of language and translation, as well as precarization related to ‘disciplinary decadence’ (Gordon, 2014, 2018). The concept ‘precarity’ derives from the French *précariat* and the Latin word *precarius;* however, what has changed or been lost as the concept has moved into the English speaking world? Furthermore, how is the term precarity (not) being used (is it even useful?) in languages beyond French and English? This also raises questions of ethics and coloniality in regard to how researchers (dis)engage with ‘Other’ languages in the production and publishing of research, recognizing the ways in which languages are tied to subjectivities, and all that can be lost in translation. Similarly, whilst the building of connections with other disciplines and knowledges is crucial for disrupting the ‘closures’ and siloization of disciplines (both from other disciplines and everyday life), we must also remain mindful of coloniality in knowledge production; namely, the power dynamics and legacies that structure borders between knowledges, along with our own positioning and complicity in these structurings (Coultas, 2022). This calls for ‘epistemic modesty’ in recognizing the limitations of our own perspectival horizons (Teo, 2019, p.31), and the development and enacting of a ‘sociohistorical intersectional consciousness’ (Fernandez et al., 2021, p.361). Additionally, we need to be ‘unsettling subjectivities of power/privilege’ and fostering ‘relationships of mutual accountability’ (Fernandez et al., 2021, p. 362), which require reflexive accountability when drawing on the work of others from a position of epistemic dominance, recognising "knowing as an activity that has material effects" (Pohlhaus, 2017, p. 41). Namely, we need to acknowledge how the nuanced meanings of ideas and concepts change as they are moved into different ontologies, epistemologies and languages.

Also methodologically, further work is needed which explores how people differentially understand, express and relate with ideas and aspects of precarity (and security). In "getting close to the grain of living on (the) edge”’ Philo and colleagues (2019; p. 2) remind us of the value of fieldwork methodologies, which invite researchers into and require them to submit to ‘deeply‐embedded, critical ethnographical encounters – quite possibly on the move (adopting “go‐alongs” of different stripes) – with their precarious… research subjects’ (p. 3). Developing methods and methodological practices that do not reinscribe colonial power relations on individuals experiencing precarity needs to be central to our research process.

### How do our findings and arguments play into broader politics of oppression, discrimination and exploitation?

In addition to considering our complicities in practices of knowledge production, it is also important to think about how the knowledge that we produce might contribute to furthering the marginalization and oppression of people living with/in precarity. What are the effects when we label someone as precarious/the precariat? How might our theorizations and formulations of ‘the social’ contribute to the moralizing and exclusion of people who do not (want to) fit into dominant framings of sociality? Likewise, in what ways might our conceptual linkages of control and security with ‘good’ psychological health and development contribute to the individualizing and anxiety‐inducing (neo)liberal logics of security (/vulnerability; Lorey, 2015)? There are no simple answers or solutions to these questions, but it is nevertheless fundamental that we as a discipline engage with them. Remaining mindful of epistemic and epistemological violences, these questions call on social psychological scholars to consider ‘the anti‐social’ (Caserio, 2006) and the ‘disaffected’ (Yao, 2021) in their work. Therefore, we invite social psychologists to think about how the knowledge produced on precarity fuels current discourses and actions that continue to precarise individuals, rather than alleviate suffering.

### How can we contribute to mobilizing the transformative potentials of precarity?

In our call for papers, we sought contributions exploring the generative and transformative potentials of precarity, such as work that develops temporariness and instability *as* praxis, or supports the building of solidarities across differences and ‘coimplication’ (Mohanty, 2003); however, these were also notably absent in the special issue submissions. The previous three reflexive questions might offer some insight into why this is. Nevertheless, Fine's epilogue (2022) provides an inspiring call for how ‘critical psychology might be otherwise’ through her discussion on ‘solidarity studies’ and the ways in which she and her colleagues are contributing to collective organizing against precarity and precarization. Solidarity building is, of course, a core aspect of community psychology, and Sonn and colleagues (2022) also implicate transnational solidarities in ‘repowering community narratives that are rooted in collective self‐determination, truth‐telling, healing, the recovery of historical memory, and the cocreation of settings from and with, to stand up for, as and with’ (p. 279). Nevertheless Fine's (2022) solidarity studies does also stand apart in how it mobilizes community research capacities with the political purpose of tackling injustices in relations with the state (e.g. health, education, immigration and law enforcement sectors).

Particularly in the UK context, we suggest that the building of solidarities between communities and social psychologists located in universities remains a challenge, related to a number of factors. First, community and critical approaches to psychology remain somewhat minoritized in UK university programmes. It would be interesting to reflect on why this is, but also more practically questions remain about how social psychologists are to develop the skills and networks necessary for working effectively and ethically with communities. Second, there is widespread community distrust of universities and academics related to legacies of transactional, extractive and exploitative experiences of research (Clark, 2008; Smith, 2021), and any work aimed at building solidarities needs to recognize and remain accountable to this backdrop. Pressures for academics to demonstrate ‘impact’, often framed in terms of how academics influence and ‘help’ communities, does little to encourage and support the reparative work that is needed (Pain et al., 2015). Lastly, questions remain over “the university's potential as an emancipatory site… in part, due to the overwhelming awareness that precarity is not just inevitable within the academy but inherent to the functioning of the ‘Academic Industrial Complex’” (Khan, 2022, p. 323 *citing* Bacchetta et al., 2018). How can scholars build meaningful relationships with communities when the majority of research teams are on temporary contracts (Albayrak‐Aydemir & Gleibs, 2022), and often do not come from, or have life experiences which are relatable for these communities (Hakim et al., 2022)? We do not say this to be pessimistic, but rather are working against any underestimation of the collective and institutionalized work that is required for the building of meaningful relationships between communities and social psychology as a field of research(ers).

## CONCLUSION

In making the case for deepening our understanding of the social psychological in existing social science research on precarity, this special issue has dialogued with precarity and worked towards intervening against aspects of precarity, with the labour of an interdisciplinary collective of authors, reviewers, editors, copy editors, and production editors. In this paper, we have highlighted the ingress that social psychology and other social science disciplines have made in understanding how precarity structures relations, is processual, is both hopeful and hopeless, and is also generatively ambivalent. We have distilled the various methodological, ontological and epistemological contributions across the nine contributions to the special issue to visibilize the dynamics of self‐other‐world relations in how precarity comes to be, and to reveal the discontinuities and disjunctures that take place when precarity is understood as a process. In developing a social psychology of precarity, we offer a double focus that uses social psychological theorizing and methods to both understand precarity *and* reflects on the roles that social psychologists play in perpetuating precarity. By asking key questions that we suggest need to be addressed in social psychological research on precarity, we offer some future directions for the field that we hope will support the steering away from disciplinary decadence and epistemological and epistemic violences, towards a social psychology of precarity that both works with/in and intervenes against ‘the precarious’.

## AUTHOR CONTRIBUTION

**Clare Coultas** co‐led in visualization, conceptualization, analysis and write‐up inclusive of drafting, reviews and editing. **Geetha Reddy** co‐led in visualization, conceptualization, analysis and write‐up inclusive of drafting, reviews and editing, and also led in the project management of the special issue as a whole. **Johanna Lukate** contributed to conceptualization, analysis and write‐up inclusive of drafting, reviews and editing, and led in the creation of Figure 1.

## CONFLICT OF INTEREST

All authors declare no conflict of interest.

## Data Availability

The data used to develop Figure 1 are not available due to legal restrictions.
